# The Galleria mellonella Infection Model Does Not Accurately Differentiate between Hypervirulent and Classical Klebsiella pneumoniae

**DOI:** 10.1128/mSphere.00850-19

**Published:** 2020-01-08

**Authors:** Thomas A. Russo, Ulrike MacDonald

**Affiliations:** a Veterans Administration Western New York Healthcare System, University at Buffalo, State University of New York, Buffalo, New York, USA; b Department of Medicine, University at Buffalo, State University of New York, Buffalo, New York, USA; c Department of Microbiology and Immunology, University at Buffalo, State University of New York, Buffalo, New York, USA; d The Witebsky Center for Microbial Pathogenesis, University at Buffalo, State University of New York, Buffalo, New York, USA; University of Missouri—Kansas City School of Medicine

**Keywords:** *Galleria mellonella* infection model, classical *Klebsiella pneumoniae*, hypervirulent *Klebsiella pneumoniae*, infection models, outbred mouse systemic infection model, virulence

## Abstract

Hypervirulent Klebsiella pneumoniae (hvKp) is an emerging pathogen of increasing concern due to its ability to cause serious organ and life-threatening infections in healthy individuals and its increasing acquisition of antimicrobial resistance determinants. Identification of hvKp is critical for patient care and epidemiologic and research studies. Five genotypic markers on the hvKp-specific virulence plasmid can accurately differentiate hvKp from the less virulent classical K. pneumoniae (cKp) strain, but it is unclear whether the possession of fewer markers accurately predicts the hvKp pathotype. Likewise, the effect, if any, of various antimicrobial resistance factors on the pathogenic potential of hvKp has been incompletely explored. The Galleria mellonella infection model is often used to assess virulence, but this tool has not been validated. Therefore, levels of lethality of defined hvKp and cKp strain cohorts were compared in *Galleria* and outbred mouse models. The murine model, but not the G. mellonella model, accurately differentiated hvKp from cKp strains. Therefore, isolates in which the pathogenic potential is ambiguous due to an incomplete hvKp biomarker profile, an incomplete pLVPK-like hvKp-specific virulence plasmid, antimicrobial resistance that could decrease biofitness, and/or the lack of a characteristic clinical presentation should be validated in an outbred murine model. These data will assist in determining the minimal genomic content needed for full expression of the hypervirulence phenotype. This information, in turn, is critical for the development of the pragmatic point-of-care testing requisite for patient care and for the performance of epidemiologic and research studies going forward.

**IMPORTANCE** Hypervirulent Klebsiella pneumoniae (hvKp) is of increasing concern because it can infect individuals in community and health care settings and because such infections are becoming difficult to treat. Identification of hvKp is important for patient care and to track its global spread. The genetic definition of hvKp, which can be used for its identification and the development of diagnostic tests, has not been optimized. Determination of possession of 4 of 5 genes that are present on the hvKp-specific virulence plasmid is highly accurate for identifying hvKp. However, an ongoing issue is whether strains that possess only some of these markers are still hypervirulent. The Galleria mellonella model and, less commonly, the murine infection model have been used to assess the virulence of these ambiguously identifiable strains. This report demonstrates that the murine model but not the G. mellonella model accurately identifies suspected hvKp strains. This information is critical for the development of diagnostics for patient care and for future research studies.

## Abstract (precis)

Hypervirulent Klebsiella pneumoniae (hvKp) is of increasing concern because it can infect individuals in community and health care settings and because such infections are becoming difficult to treat. Identification of hvKp is important for patient care and to track its global spread. The genetic definition of hvKp, which can be used for its identification and the development of diagnostic tests, has not been optimized. Determination of possession of 4 of 5 genes that are present on the hvKp-specific virulence plasmid is highly accurate for identifying hvKp. However, an ongoing issue is whether strains that possess only some of these markers are still hypervirulent. The Galleria mellonella model and, less commonly, the murine infection model have been used to assess the virulence of these ambiguously identifiable strains. This report demonstrates that the murine model but not the G. mellonella model accurately identifies suspected hvKp strains. This information is critical for the development of diagnostics for patient care and for future research studies.

## INTRODUCTION

Hypervirulent Klebsiella pneumoniae (hvKp) is an evolving global pathotype that is more virulent than classical K. pneumoniae (cKp). In contrast to the usual health care-associated epidemiology of cKp infections, hvKp has the ability to cause serious infection in otherwise healthy individuals in community settings (1). hvKp frequently presents with multiple sites of infection and has an increased ability to cause central nervous system infection, necrotizing fasciitis, and endophthalmitis compared to cKp and other pathogens in the family *Enterobacteriaceae*, requiring rapid recognition and site-specific treatment (1). Similarly to cKp, hvKp strains are becoming increasingly resistant to antimicrobials via acquisition of conjugal plasmids carrying resistance determinants or in cases in which an extensively drug-resistant (XDR) cKp strain acquires hvKp-specific virulence genes (2–4). Of concern, hvKp infections are becoming increasingly prevalent in the health care setting (5). Needless to say, the confluence of hypervirulence and XDR in a pathogen that can infect all patient populations is alarming.

The ability to accurately discriminate between hvKp and cKp is needed to optimize patient care and infection control practices, to track global epidemiology and antimicrobial resistance trends, to enable clinical studies, and to delineate virulence factors for the development of countermeasures (6). To those ends, the use of five genotypic markers (*peg-344*, *iroB*, *iucA*, *rmpA*, and *rmpA2*) present on the hvKp virulence plasmid and >30 μg/ml of total siderophore production was shown in an epidemiologic analysis to provide >0.95 diagnostic accuracy for differentiating hvKp from cKp (7). Importantly, these biomarkers were experimentally validated in an outbred murine systemic infection model (7). However, nearly all of the hvKp strains studied in that report were isolated from patients with a clinical presentation consistent with hvKp infection and possessed all 5 genotypic markers. The minimal genomic content needed for full expression of the hypervirulence phenotype and whether a spectrum of virulence exists remain unclear. A number of studies have reported variants that have been presumptively designated to be hvKp by virtue of possessing only some of the defining hvKp genotypic markers (*peg-344*, *iroB*, *iucA*, *rmpA*, and *rmpA2*) and/or a portion of the canonical hvKp virulence plasmid pLVPK (3). Further, some isolates that contain some or all of these markers express antimicrobial resistance by various mechanisms. However, the overall effect of this variable genomic content and of the expression of various antimicrobial resistance determinants on the virulence of hvKp strains has been incompletely explored. Although clinical data could lend insights into the pathogenic potential of these strains, in many cases, these data were unavailable (8), the strain in question was a colonizer (4), or the discriminatory power of the clinical features of infection was obscured by the underlying host status (3). Therefore, the relative degree of hypervirulence possessed by many of these strains is unclear. Currently, studies in outbred murine infection models represent the best experimental tools to assist in defining the pathogenic potential of these strains. However, many investigators have utilized *in vitro* neutrophil-mediated bactericidal assay or virulence in the Galleria mellonella infection model or both as alternatives to validate the hypervirulence phenotype of these ambiguous strains. Salient reasons include decreased costs and the use of a less extensively sentient genus that does not require institutional review board (IRB) approval. However, cKp strains can also be resistant to neutrophil-mediated killing (9) and the accuracy of the *Galleria* model for differentiating hvKp from cKp has not been validated in large, defined hvKp and cKp strain cohorts. Therefore, the goal of this report was to assess the accuracy of the G. mellonella model as a tool for validating the hypervirulence phenotype in K. pneumoniae by comparing well-characterized hvKp and cKp strain cohorts. The hvKp strain cohort exhibited a small, dose-dependent increase in virulence compared the cKp strain cohort on a population basis, but significant overlap was observed at the individual strain level. These data demonstrate that the G. mellonella model cannot be used to accurately define hvKp.

## RESULTS

### The outbred murine infection model was highly accurate for differentiating the hvKp and cKp strain cohorts.

**FIG 1:**
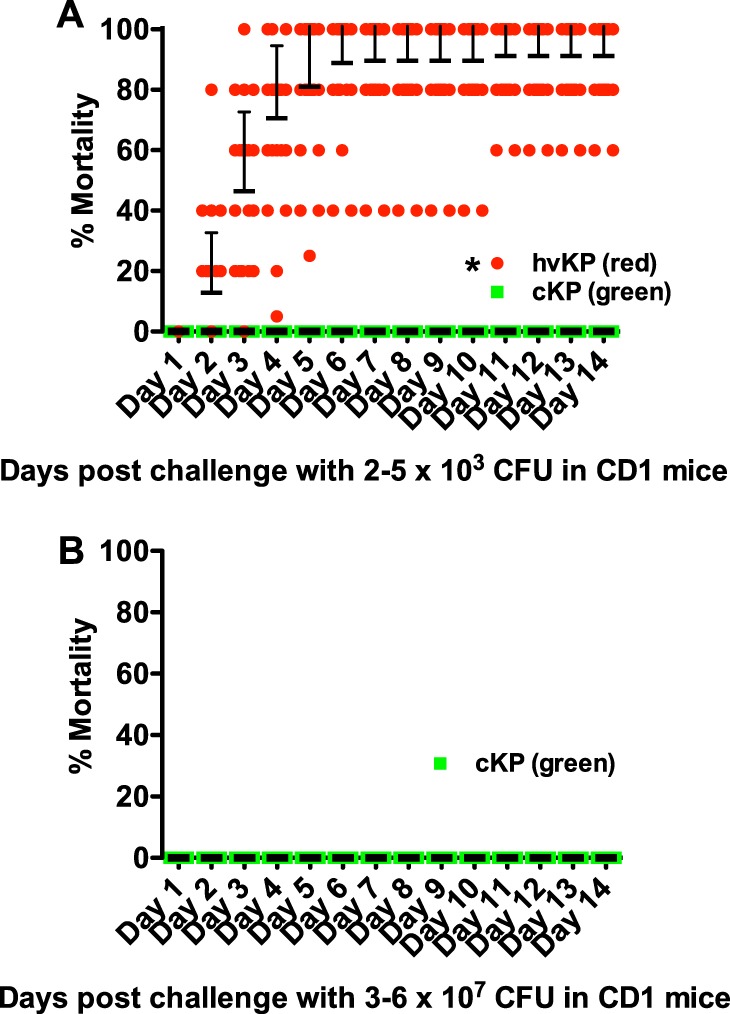
Mortality in the CD1 murine infection models after challenge with the hvKp and cKp strain cohorts. Each symbol represents the mean mortality for a given strain over time. *n* = 25 for each strain cohort. (A) Day 1 to day 14 mortality after challenge with 2 × 10^3^ to 5 × 10^3^ CFU of both the hvKp and cKp strain cohorts. For the hvKp strain cohort, means ± standard deviations (SD) are depicted. (B) Murine model: day 1 to day 14 mortality after challenge with the cKp strain cohort at challenge inocula of 3 × 10^7^ to 6 × 10^7^ CFU. Animals were not challenged with the hvKp strain cohort at this challenge inoculum due to the predicted lethal result. *, *P* < 0.05 (2-way ANOVA with a Bonferroni posttest).

The mean 14-day death rates for the hvKp and cKp strain cohorts were 91.2% (median, 100%; lower and upper 95% confidence interval, 85.8 and 96.6) and 0%, respectively, with challenge inocula of 2 × 10^3^ to 5 × 10^3^ CFU (* P <* 0.05) (Fig. 1A). Further, the mean 14-day death rate for cKp strain cohort remained at 0% despite the use of 4-log-higher challenge inocula of 3 × 10^7^ to 6 × 10^7^ CFU; mice were spared challenge with the hvKp strain cohort at these challenge inocula due to the predicted lethal outcome (* P <* 0.05) (Fig. 1B). These data demonstrate that this model can accurately differentiate canonical hvKp strains that express a complete hypervirulent genotype from canonical cKp strains that do not possess any hvKp-specific genotypic biomarkers over a range of challenge inocula of at least 4 logs, a biologically highly significant difference.

### A significant overlap in virulence was observed in the G. mellonella infection model.

**FIG 2:**
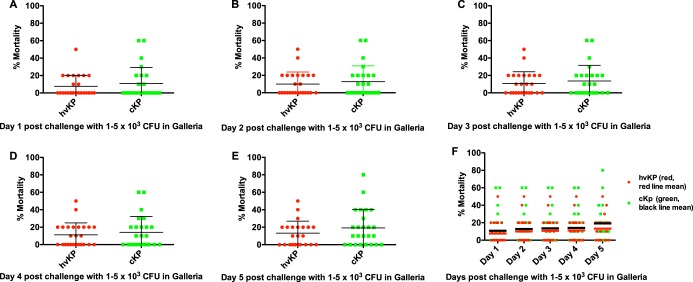
Mortality in the Galleria mellonella infection model after challenge with the hvKp and cKp strain cohorts at inocula of 1 × 10^3^ to 5 × 10^3^ CFU. Each symbol represents the mean mortality for a given strain. *n* = 25 for each strain cohort. (A) Day 1. (B) Day 2. (C) Day 3. (D) Day 4. (E) Day 5. (F) Days 1 to 5. For panels A to E, means ± SD are depicted; for panel F, the horizontal line depicts the mean.

**FIG 3:**
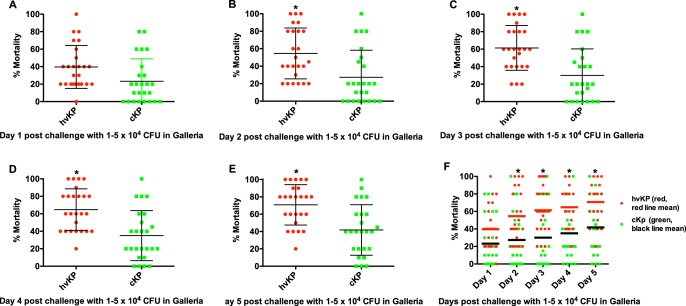
Mortality in the Galleria mellonella infection model after challenge with the hvKp and cKp strain cohorts at inocula of 1 × 10^4^ to 5 × 10^4^ CFU. Each symbol represents the mean mortality for a given strain. *n* = 25 for each strain cohort. (A) Day 1. (B) Day 2. (C) Day 3. (D) Day 4. (E) Day 5. (F) Days 1 to 5. For panels A to E, means ± SD are depicted; for panel F, the horizontal line depicts the mean. *, *P* < 0.05 (2-way ANOVA with a Bonferroni posttest).

**FIG 4:**
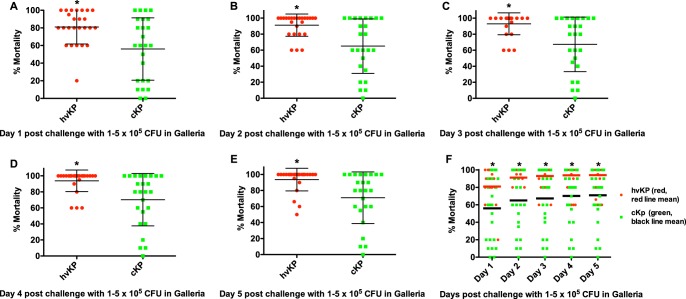
Mortality in the Galleria mellonella infection model after challenge with hvKp and cKp strain cohort inocula of 1 × 10^5^ to 5 × 10^5^ CFU. Each symbol represents the mean mortality for a given strain. *n* = 25 for each strain cohort. (A) Day 1. (B) Day 2. (C) Day 3. (D) Day 4. (E) Day 5. (F) Days 1 to 5. For panels A to E, means ± SD are depicted; for panel F, the horizontal line depicts the mean. *, *P* < 0.05 (2-way ANOVA with a Bonferroni posttest).

Pilot experiments were performed using challenge inocula of 1 × 10^2–6^ to 5 × 10^2–6^ to identify the bacterial titers that held the greatest promise for discrimination between strain cohorts. Challenge inocula of 1 × 10^2^ to 5 × 10^2^ and 1 × 10^6^ to 5 × 10^6^ CFU resulted in nearly 0% and 100% lethality, respectively, regardless of whether the challenge strain was an hvKp or a cKp isolate; therefore, challenge inocula of 1 × 10^3^ to 5 × 10^3^ (Fig. 2), 1 × 10^4^ to 5 × 10^4^ (Fig. 3), and 1 × 10^5^ to 5 × 10^5^ CFU (Fig. 4) were utilized for further study. The mean 5-day death rates for the hvKp and cKp strain cohorts after challenge with inocula of 1 × 10^3^ to 5 × 10^3^ CFU were 13.2% (median, 10%; lower and upper 95% confidence interval, 7.5 and 18.8) and 23.2% (median, 20%; lower and upper 95% confidence interval, 13 and 33.4), respectively (*P > *0.05). The mean 5-day death rates for the hvKp and cKp strain cohorts after challenge with inocula of 1 × 10^4^ to 5 × 10^4^ CFU were 67.2% (median, 60%; lower and upper 95% confidence interval, 56.9 and 77.5) and 41.4% (median, 40%; lower and upper 95% confidence interval, 29.6 and 53.2), respectively (*P* < 0.05). The mean 5-day death rates for the hvKp and cKp strain cohorts after challenge with inocula of 1 × 10^5^ to 5 × 10^5^ CFU were 93.4% (median, 100%; lower and upper 95% confidence interval, 87.4 and 99.4) and 71% (median, 80%; lower and upper 95% confidence interval, 57.7 and 84.3), respectively (*P < *0.05) (Fig. 2, 3, and 4) (Table 1). Although the 5-day death rate for hvKp strain cohort was statistically significant using the 1 × 10^4^ to 5 × 10^4^ CFU and 1 × 10^5^ to 5 × 10^5^ CFU challenge inocula compared to the cKp strain cohort, the differences were small (25.8% and 22.4%, respectively). No statistically significant difference was observed between strain cohorts with 1 × 10^3^ to 5 × 10^3^ CFU challenge inocula. Further, comparisons between individual strains revealed a significant overlap in death rates (Fig. 2, 3, and 4). Therefore, the G. mellonella model was unable to accurately differentiate canonical hvKp strains that express a complete hypervirulent genotype from canonical cKp strains that do not possess any hvKp-specific genotypic biomarkers.

**TABLE 1:** Daily percent mortality in the Galleria mellonella infection model

Challenge inoculum and day of challenge	% mortality (mean; median; lower, upper 95% confidence interval) with:	
	hvKp strain cohort	cKp strain cohort
1 × 10^3^–5 × 10^3^ CFU		
Day 1	7.6; 0.0; 2.5, 12.7	14; 0.0; 4.0, 24
Day 2	10; 0.0; 4.3, 15.7	16; 0.0; 6.2, 25.8
Day 3	10.8; 10; 5.2, 16.4	16.8; 10; 7.1, 26.5
Day 4	11.2; 10; 5.8, 16.8	17.2; 10; 7.4, 27
Day 5	13.2; 10; 7.5, 18.8	23.2; 20; 13, 33.4

1 × 10^4^–5 × 10^4^ CFU		
Day 1	39.6; 40; 29.5, 49.7	23.2; 20; 12.6, 33.8
Day 2	54.6; 45; 42.6, 66.6	27.4; 20; 14.7, 40.1
Day 3	61.4; 60; 50.8, 72	30; 20; 17.5, 42.5
Day 4	62.4; 60; 52, 72.8	35; 20; 23.2, 46.8
Day 5	67.2; 60; 56.9, 77.5	41.4; 40; 29.6, 53.2

1 × 10^5^–5 × 10^5^ CFU		
Day 1	80.6; 80; 72.4, 88.8	56; 60; 41.4, 70.6
Day 2	90.8; 100; 84.7, 97	65; 60; 51, 79
Day 3	92.6; 100; 86.5, 98.7	67.4; 80; 53.4, 81.5
Day 4	93.4; 100; 87.4, 99.4	70.2; 80; 56.7, 83.7
Day 5	93.4; 100; 87.4, 99.4	71; 80; 57.7, 84.3

## DISCUSSION

These data demonstrate that the widely used G. mellonella infection model failed to accurately differentiate between canonical representatives of the hvKp and cKp pathotypes. The reason(s) for the poor performance of the G. mellonella model is unclear. Both mice and *Galleria* possess an innate host defense system (10) known to protect against *Klebsiella* infection, at least in part (9, 11–13). Perhaps hvKp-specific virulence factors (e.g., increased production of capsule and siderophores) are critical for growth and survival in mice but not in *Galleria*. Further, a possible misconception with respect to the validity of the *Galleria* model for use in discriminating between hvKp and cKp can be seen in examination of data in Fig. 2, 3, and 4. Selected cKp strains demonstrate decreased virulence in the *Galleria* model. However, the use of these strains as a control could be deceiving if a more virulent strain(s) is designated hvKp. Numbers of both cKp and hvKp strains showed increased virulence, thereby demonstrating the poor discriminatory power of this model for these pathotypes.

In this study, in which canonical hvKp and cKp strains were studied, the outbred murine infection model proved to be highly discriminatory with respect to differentiation of these groups. However, it is likely that some cKp and hvKp strains may possess more or less pathogenic potential than the strains studied. It is predicted that the G. mellonella model would perform even less well in differentiating these more ambiguous pathotypes. In contrast, the ability of the murine model to unequivocally differentiate between the canonical hvKp and cKp strain cohorts over at least a 4-log challenge inocula window with 100% accuracy makes this model ideal for defining the relative pathogenic potential of the strains possessing the more ambiguous pathotypes.

The ability to accurately distinguish an hvKp from a cKp pathotype has important implications for patient care. Identification of an hvKp strain would alert the clinician to seek occult sites of infection that could require source control and/or a site-specific modification of antimicrobial therapy. Other potential implications include the need for a more prolonged treatment course and consideration of infection control measures. Of course, controlled data are welcomed to fill knowledge gaps for several hvKp management issues, but the ability to accurately identify hvKp is needed to perform such trials.

Published data from our group demonstrated that *iroB*, *peg-344*, *iucA*, *rmpA*, and *rmpA2* are highly accurate biomarkers for identifying hvKp. However, in that previous study, all of the strains identified as hvKp possessed 4 or 5 of these markers. An ongoing issue is whether possession of fewer markers still accurately predicts the hvKp pathotype. Likewise, the effect, if any, of various antimicrobial resistance determinants on the pathogenic potential of hvKp has been incompletely explored. Our data demonstrate that the outbred murine model, but not the *Galleria* model, would enable these issues to be resolved. This information is critical for the development of pragmatic point of care testing and for the performance of epidemiologic and research studies.

One limitation is that our results are presently applicable only to hvKp and cKp strains. Further, it is unclear whether the use of inbred mice or different bacterial challenge routes would be as discriminatory as the use of outbred mice challenged subcutaneously.

Our data demonstrate that an outbred mouse model, but not the G. mellonella model, is appropriate for validating suspected hvKp strains. This murine model will be particularly useful for studying strains in which the pathogenic potential is ambiguous due to an incomplete hvKp biomarker profile (7) or pLVPK-like hvKp-specific virulence plasmid, antimicrobial resistance that could decrease biofitness, and/or the lack of a characteristic clinical presentation.

## MATERIALS AND METHODS

### Strain cohorts.

Each of the cohorts, all of which were obtained from geographically diverse regions, consisted of 25 unambiguous hvKp and cKp strains as defined by genomic markers, quantitative siderophore production, and lethality in an outbred murine model (see Table S1 in the supplemental material). The hvKp strains were blood or liver aspirate isolates, and all of the cKp strains were blood isolates. Lethality results were previously reported qualitatively for these strains (see Table S2 in reference 7) and are reported quantitatively in this report. For strain assessment in infection models, bacteria from a freshly streaked plate were grown overnight in 1 ml of lysogeny broth at 120 rpm in a shaking water bath at 37°C. In the morning, the overnight cultures underwent 10-fold serial dilutions in 1× phosphate-buffered saline (1× PBS) and were kept on ice until use. For bacterial enumeration, 100 μl of the 10^−6^ and 10^−7^ serial dilutions were plated on LB agar in duplicate and were incubated overnight at 37°C; 96% were within the reported ranges of 1 × 10^3^ to 5 × 10^3^, 1 × 10^4^ to 5 × 10^4^, and 1 × 10^5^ to 5 × 10^5^ CFU for the G. mellonella infection model (two strains had slightly lower challenge inocula, namely, 8.5 × 10^2–4^ for hvKp11 and 9.0 × 10^2–4^ for cKp2); 100% were within the reported range of 2 × 10^3^ to 5 × 10^3^ CFU (for both the hvKp and cvKp strain cohorts) and 3 × 10^7^ to 6 × 10^7^ CFU (for the cKp strain cohort) for the murine infection model.

10.1128/mSphere.00850-19.1 TABLE S1 hvKp and cKp strain cohorts. Download Table S1, XLSX file, 0.02 MB. Copyright © 2020 Russo and MacDonald. 2020 Russo and MacDonald This content is distributed under the terms of the Creative Commons Attribution 4.0 International license.

### G. mellonella infection model.

Larvae of the wax moth G. mellonella were treated with a juvenilizing agent for the prevention of webbing and cocoons (SpeedyWorm [wax worms]). The day before infection, healthy, cream-colored larvae between 150 and 200 mg in body weight were placed in a plastic container with air holes and left at room temperature. The next morning, 5 larvae per challenge inoculum were placed in a petri dish and health was assessed by observing whether the larvae were able to quickly roll over when placed on their back. The petri dish was placed on ice for 2 to 3 min to temporarily immobilize the larvae before infection. Challenge inocula of 1 × 10^3^ to 5 × 10^3^, 1 × 10^4^ to 5 × 10^4^, and 1 × 10^5^ to 5 × 10^5^ CFU were administered in 10-μl volumes through the left second hind proleg (or through an alternative if needed) using a custom-made 29-gauge removable needle (RN) (12.5-mm, 30° needle) and a 10-μl gas-tight Hamilton syringe (Hamilton Company). The syringe was submerged in 70% ethanol for 2 to 3 min, rinsed 5 times with 70% ethanol, and then rinsed 5 times with sterile 1× PBS between strain procedures. After challenge, larvae were placed in a petri dish and incubated at 37°C for 5 days. Mortality was assessed daily. Pilot studies of 2 to 3 independent sets of 5 larvae for a given strain and challenge inoculum demonstrated that results were reproducible between sets; therefore, a set of *n* = 5 was used for most strains for each challenge inoculum. Forty control larvae received 10 μl of 1× PBS; a 5% 5-day mortality rate was observed in this group.

### Outbred murine infection model.

Studies were reviewed and approved by the University at Buffalo and Veterans Administration Institutional Animal Care Committees. This study was done in strict accordance with the recommendations in the Guide for the Care and Use of Laboratory Animals of the National Institutes of Health; all efforts were made to minimize suffering. CD1 mice were challenged subcutaneously with challenge inocula of 2 × 10^3^ to 5 × 10^3^ for both the hvKp and cKp strain cohorts or with challenge inocula of 3 × 10^7^ to 6 × 10^7^ CFU for the cKp strain cohort in 100 μl of 1× PBS and observed for 14 days for the development of an in extremis state or death (7).

### Statistical analyses.

Statistical analyses were performed using GraphPad Prism 5.0. Two-way analysis of variance (ANOVA) was performed to analyze *in vivo* infection model data with the cKp and hvKp strain cohorts as the first variable and duration of infection as the second variable. Bonferroni posttests were used to account for multiple comparisons. A *P* value of <0.05 was considered statistically significant.
